# FMRP Mediates mGluR_5_-Dependent Translation of Amyloid Precursor Protein

**DOI:** 10.1371/journal.pbio.0050052

**Published:** 2007-02-13

**Authors:** Cara J Westmark, James S Malter

**Affiliations:** Department of Pathology and Laboratory Medicine, Waisman Center for Developmental Disabilities, University of Wisconsin, Madison, Wisconsin, United States of America; Massachusetts Institute of Technology, United States of America

## Abstract

Amyloid precursor protein (APP) facilitates synapse formation in the developing brain, while beta-amyloid (Aβ) accumulation, which is associated with Alzheimer disease, results in synaptic loss and impaired neurotransmission. Fragile X mental retardation protein (FMRP) is a cytoplasmic mRNA binding protein whose expression is lost in fragile X syndrome. Here we show that FMRP binds to the coding region of APP mRNA at a guanine-rich, G-quartet–like sequence. Stimulation of cortical synaptoneurosomes or primary neuronal cells with the metabotropic glutamate receptor agonist DHPG increased APP translation in wild-type but not *fmr-1* knockout samples. APP mRNA coimmunoprecipitated with FMRP in resting synaptoneurosomes, but the interaction was lost shortly after DHPG treatment. Soluble Aβ_40_ or Aβ_42_ levels were significantly higher in multiple strains of *fmr-1* knockout mice compared to wild-type controls. Our data indicate that postsynaptic FMRP binds to and regulates the translation of APP mRNA through metabotropic glutamate receptor activation and suggests a possible link between Alzheimer disease and fragile X syndrome.

## Author Summary

Alzheimer disease (AD) and fragile X syndrome (FXS) are devastating neurological disorders associated with synaptic dysfunction resulting in cognitive impairment and behavioral deficits. Despite these similar endpoints, the pathobiology of AD and FXS have not previously been linked. We have established that translation of amyloid precursor protein (APP), which is cleaved to generate neurotoxic βamyloid, is normally repressed by the fragile X mental retardation protein (FMRP) in the dendritic processes of neurons. Activation of a particular subtype of glutamate receptor (mGluR_5_) rapidly increases translation of APP in neurons by displacing FMRP from a guanidine-rich sequence in the coding region of APP mRNA. In the absence of FMRP, APP synthesis is constitutively increased and nonresponsive to mGluR-mediated signaling. Excess APP is proteolytically cleaved to generate significantly elevated βamyloid in multiple mutant mouse strains lacking FMRP compared to wild type. Our data support a growing consensus that FMRP binds to guanine-rich domains of some dendritic mRNAs, suppressing their translation and suggest that AD (neurodegenerative disorder) and FXS (neurodevelopmental disorder) may share a common molecular pathway leading to the overproduction of APP and its protein-cleaving derivatives.

## Abstract 3

FMRP, the cytoplasmic mRNA-binding protein lost in fragile X syndrome, regulates the translation of amyloid precursor protein in neurons.

## Introduction

Alzheimer disease (AD) is a neurodegenerative disorder characterized by senile plaques and neurofibrillary tangles. The plaques are predominantly composed of beta-amyloid (Aβ), a 39–42 amino acid peptide cleaved from the amyloid precursor protein (APP). APP is likely important for synapse formation in the developing brain [1], while excess Aβ causes impaired synaptic function [2]. Disordered synaptic transmission is also a hallmark of other neuronal disorders, such as epilepsy and fragile X mental retardation syndrome (FXS).

FXS is the most prevalent form of inherited mental retardation, affecting one in 4,000 men and one in 8,000 women. This X chromosome–linked disorder is characterized by moderate to severe mental retardation (overall IQ <70), autistic-like behavior, seizures, facial abnormalities (large, prominent ears and long, narrow face) and macroorchidisim [3]. At the neuroanatomic level, FXS is distinguished by an overabundance of long, thin, tortuous dendritic spines with prominent heads and irregular dilations [4,5]. The increased length, density, and immature morphology of dendritic spines in FXS suggest an impairment of synaptic pruning and maturation.

In the majority of cases, FXS results from a trinucleotide (CGG) repeat expansion to >200 copies in the 5′-UTR of the *fmr-1* gene (located at Xq27.3) [6]. The CGG expansion is associated with hypermethylation of the surrounding DNA, chromatin condensation, and subsequent transcriptional silencing of the *fmr-1* gene, resulting in the loss of expression of fragile X mental retardation protein (FMRP) [7].

FMRP is an mRNA-binding protein that is ubiquitously expressed throughout the body, with significantly higher levels in young animals [8]. The protein has two heterogeneous nuclear ribonucleoprotein (hnRNP) K homology domains and one RGG box as well as nuclear localization and export signals. FMRP interacts with BC1 RNA as well as a number of RNA-binding proteins, including nucleolin and YB1 and the FMRP homologs FXR1 and FXR2 [9]. FMRP has been implicated in translational repression [10–15], and in the brain, cosediments with both translating polyribosomes [16] and with mRNPs [12]. The RGG box of FMRP binds to intramolecular G quartet sequences in target mRNAs [17], while the KH2 domain has been proposed to bind to so-called kissing complex RNAs based on in vitro selection assays [18]. In addition, FMRP binds to uridine-rich mRNAs [19,20]. In aggregate, more than 500 mRNA ligands for FMRP have been identified, many with the potential to influence synaptic formation and plasticity [10,17].

FMRP is required for type 1 metabotropic glutamate receptor (mGluR)–dependent translation of synaptic proteins, including FMRP and postsynaptic density 95 (PSD-95) [21,22]. Both PSD-95 and FMRP mRNAs contain putative G-quartets in their 3′-UTR and coding sequence, respectively [22,23]. Database searches revealed that APP mRNA possesses a G-quartet–like motif in the coding region (position 825–846 of the mouse sequence) embedded within a guanine-rich domain (694–846) containing several DWGG repeats. APP mRNAs (70% of APP695 and 50% of APP751/770) are associated with polyribosomes in rat brain [24], suggesting that translational regulation could play an important role in APP production. Indeed, APP contains a 5′-UTR iron response element previously implicated in translation control [25]. Therefore, we hypothesized that APP mRNA translation would be regulated by FMRP.

We now show that after stimulation with the mGluR agonist DHPG, APP levels increased significantly in wild-type (WT) but not synaptoneurosomes (SNs) or cultured neurons from knockout (KO) animals. In KO SNs or neurons, APP was constitutively elevated. APP mRNA coimmunoprecipitated with FMRP in WT, resting SNs, but this interaction was lost with DHPG treatment. FMRP monomer bound to the 5′ end of the G-rich sequence in the coding region of APP mRNA. Our data indicate that FMRP represses the translation of APP through mGluR-dependent interactions with APP mRNA. Consistent with constitutively elevated APP levels, the proteolytic products Aβ_40_ and Aβ_42_ are elevated in the brains of *fmr-1* KO mice compared to WT.

## Results

### APP mRNA Coimmunoprecipitates with Anti-FMRP

**Figure 1:**
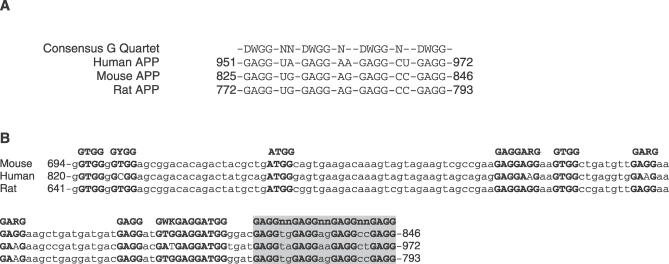
The Coding Region of APP mRNA Contains a Putative G-Quartet Sequence within a G-Rich Region Containing Several DWGG Repeats (A) Alignment of the canonical G-quartet motif with the putative G-quartet sequence in human, mouse, and rat APP mRNAs. (B) Alignment of the G-rich region of the human, mouse, and rat APP genes. The predicted G-quartet sequence is located at position 825–846 of the mouse gene and is highlighted.

Our laboratory has shown that FMRP and PSD-95 mRNAs are rapidly translated in mouse primary cortical neurons in response to the type 1 mGluR agonist DHPG [22]. Normal regulation was lost in *fmr-1* KO-derived neurons, implicating FMRP in this process. These and other FMRP-regulated mRNAs contain G-quartets, which have been proposed as at least one site of mRNA/FMRP interaction [17]. Database searches of brain mRNAs revealed that the coding region of human, mouse, and rat APP mRNAs contained a G-quartet–like sequence (Figure 1A) within a G-rich domain containing several DWGG repeats (Figure 1B). The putative G-quartet motif in APP mRNA has the potential to form a stable structure containing three guanine planes (Figure S1). FMRP binds to G-rich sequences (so-called G-quartets; consensus site: DWGG-N_(0–2)_-DWGG-N_(0–1)_-DWGG-N_(0–1)_-DWGG, where D is any nucleotide except C and W is A or U) [17] arranged in a planar conformation and stabilized by Hoogsteen-type hydrogen bonds. In the human APP mRNAs, the G-rich region containing the putative G-quartet motif is found in all three splice variants (APP695, APP751, and APP770; 87 nucleotides upstream of the sequence coding for the Kunitz-type protease inhibitor domain, which is missing in APP695). FMRP also binds to kissing complex sites [18], but APP mRNA lacks such a site. Therefore, we prepared cortical lysates as well as SNs from WT mice and immunoprecipitated FMRP. Contrary to a previous report utilizing a different protocol for the preparation of SNs [26], APP mRNA is present in SNs, and reverse transcription (RT)–PCR revealed that the message was brought down with specific, but not control, antisera in cortical lysates as well as SNs (unpublished data). Thus, APP mRNA is a potential target of FMRP, presumably via the coding region putative G-quartet.

### FMRP Regulates APP Translation

**Figure 2:**
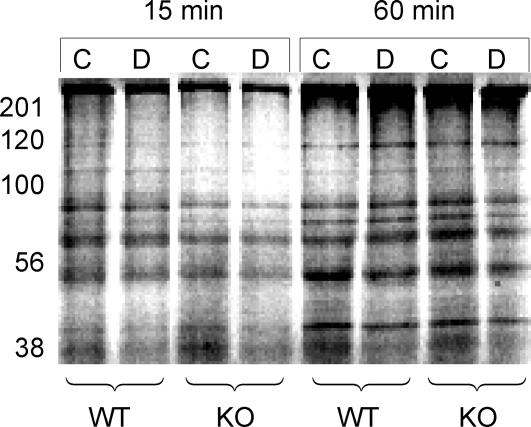
SNs Prepared from WT and *fmr-1* KO Cortices Are Translationally Active SDS-PAGE analysis of ^35^S-Met–labeled SNs without (C lanes) or with (D lanes) DHPG stimulation for times shown in minutes. The data are representative of multiple experiments: *n =* 6 (WT); *n =* 5 (KO).

APP is highly expressed in neurons and dendrites and may promote synaptic maturation [1]. Conversely, overexpression of APP and its proteolytic product, Aβ, have been implicated in the synaptic losses seen early in the development of AD [27]. Therefore, we asked if APP translation was regulated by dendritic FMRP. We utilized *fmr-1* KO mice, a rodent model for FXS, that display dendritic spine anomalies similar to that in the human disorder [28–30]. Cortical SNs were prepared from both WT and *fmr-1* KO mice, and overall protein synthesis was analyzed in response to DHPG (100 μM)–induced mGluR activation. SNs from either animal were metabolically active with equivalent total ^35^S-Met incorporation (Figure 2). Therefore, FMRP was not required for basal protein synthesis, which is in agreement with a prior report [31]. However, we did not observe an increase in overall protein synthesis in response to DHPG, whereas Weiler and colleagues [31] observed a 1.3-fold increase in ^35^S-Met incorporation after 5 min of stimulation.

**Figure 3:**
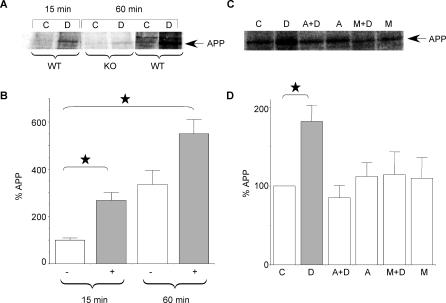
mGluR Activation Increases APP Translation in SNs (A) Immunoprecipitated, ^35^S-labeled APP (120-kDa band) from WT (15 min) and KO and WT (60 min) SNs analyzed by SDS-PAGE and (B) plotted as a percentage of APP synthesis; *n =* 3 repetitions. Asterisk indicates significant differences, with *p* = 0.008 between ±DHPG samples at 15 min and *p* = 0.016 between control at 15 min and DHPG at 60 min. For the control samples at 15 and 60 min, *p* = 0.056, and for the samples with or without DHPG at 60 min, *p* = 0.05. (C) Immunoprecipitated, ^35^S-labeled APP (120-kDa band) from WT SNs treated with DHPG, anisomycin, and MPEP, analyzed by SDS-PAGE, and (D) plotted as a percentage of APP synthesis; *n =* 3 repetitions (DHPG), *n =* 4 (anisomycin + DHPG and anisomycin), and *n* = 5 (MPEP + DHPG and MPEP).

To assess de novo synthesis, ^35^S-labeled WT or KO SNs were immunoprecipitated with anti-APP. After 15 min of incubation, untreated WT SNs translated modest amounts of APP, which rapidly increased by 2.7-fold with DHPG treatment. After 1 hr, APP remained elevated in stimulated SNs over the control, but the difference was less (1.6-fold) than at 15 min, suggesting more persistent translation in the unstimulated controls, slowing of new synthesis after stimulation, and/or compensatory protein turnover in the DHPG-treated samples (Figure 3A and 3B). In KO SNs, APP synthesis was less than in WT SNs and showed a minimal response to DHPG. The translational inhibitor anisomycin blocked DHPG-mediated synthesis of APP, as did the specific mGluR_5_ inhibitor MPEP (Figure 3C and 3D).

**Figure 4:**
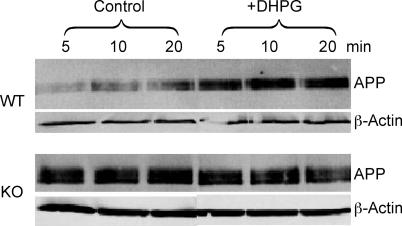
Differential Regulation of APP Levels in WT and KO SNs Western blots of WT (top panel) and KO (bottom panel) SN treated with or without DHPG (5, 10, and 20 min) and hybridized with anti-APP and anti–β-actin antibodies. The data are representative of three experiments, and quantitation with ImageQuant software demonstrates a 1.6–1.8-fold increase in APP between untreated and DHPG-stimulated WT SNs at all of the times tested.

In order to assess changes in steady-state levels, rather than new protein synthesis, APP was measured in WT and KO SNs in response to DHPG by Western blot analysis (Figure 4). In WT SNs, there was a rapid increase in total APP levels within 5 min of DHPG treatment (1.6-fold, *n =* 3), which was completely absent in KO SNs. Regardless of treatment, APP levels remained nearly constant over time in KO SNs, as did β-actin. In the absence of DHPG, steady-state levels of APP were substantially higher in KO SNs compared to WT SNs. Within 20 min of DHPG treatment, APP levels in WT SNs approached those seen in unstimulated KO SNs (Figure 4). Protease inhibitors increased steady-state levels of APP in WT SNs to those seen in KO SNs (unpublished data). These data suggest that APP mRNA is translationally repressed by FMRP in unstimulated WT SNs. mGluR activation rapidly derepresses APP synthesis as shown for FMRP and PSD-95 [21,22]. APP levels during maximal derepression approach those seen constitutively in *fmr-1* KO cells. After the cessation of mGluR signaling, APP levels presumably drop due to degradation, which appears more robust in WT than KO cells.

### FMRP Regulates Dendritic APP Levels in Cultured Neurons

**Figure 5:**
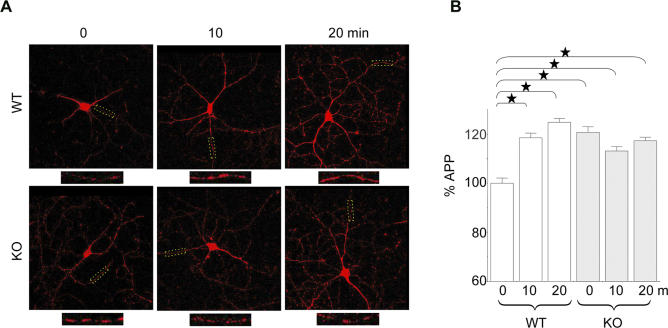
DHPG Enhances APP Translation in WT but Not *fmr-1* KO Neurons (A) Immunofluorescent confocal images of WT (top) and KO (bottom) neuronal cells treated with or without DHPG (0, 10, and 20 min) and hybridized with anti-22C11 APP primary and anti-mouse rhodamine-conjugated secondary antibodies. The dashed yellow rectangles encompass segments of dendrites, which are enlarged and displayed below the photos. (B) Dendritic APP levels were quantitated with ImageJ software and plotted as a percentage of untreated WT samples. Asterisks indicate significant differences, with *p* < 0.001 between the pairs.

SNs are a relatively crude preparation of pre- and postsynaptic densities that are contaminated with other cell types, such as astrocytes, which form synapses with neurons. Thus, we prepared primary embryonic day–18 cortical neuron cultures from WT and *fmr-1* KO brains and assessed dendritic APP levels by immunofluorescence. APP was found in the cell body as well as dendritic puncta of both WT and *fmr-1* KO neurons (Figure 5A). There was a 21% increase in the basal level of APP in untreated *fmr-1* KO neurons compared to WT (Figure 5B). Neurons stimulated with DHPG for 10 and 20 min prior to cell fixation showed a 18%–25% increase in dendritic APP levels in WT but no increase in *fmr-1* KO cultures (Figure 5B). These data confirm our findings in SNs that (1) *fmr-1* KO mice have higher basal synaptic levels of APP, and (2) DHPG increases APP levels selectively in WT samples. These data also demonstrate that FMRP and mGluR activation regulate APP synthesis in both FVB and C57BL/6 mice, as the SNs were prepared from the former strain, and the primary cortical neurons from the latter strain.

### mGluR Activation Does Not Affect APP mRNA Stability

FMRP and homologs have been implicated in the control of mRNA decay. There are increased APP mRNA levels in the cerebral cortex, hippocampus, and cerebellum in a FXS mouse model [32], and FXR1P, an FMRP homolog, is an AU-rich element–binding protein that binds to and regulates TNFα mRNA stability and translation [33]. APP mRNA contains two 3′-UTR *cis*-elements within 200 bases of the stop codon that mediate message stability. Hence, we analyzed APP mRNA and 18S rRNA decay in SNs by real-time PCR. APP mRNA did not decay over 120 min regardless of mGluR activation in WT and KO SNs (Figure S2). These data indicate that mGluR-dependent APP translation was independent of mRNA stabilization. APP mRNA has a half-life of approximately 5 h in resting immune cells, which is prolonged in activated cells [34,35] or rat PC12 (Westmark and Malter, unpublished data). Thus, APP mRNA decays with comparable kinetics in SNs and mammalian cells.

### mGluR Activation Dislodges APP mRNA from an mRNP Complex Containing FMRP

**Figure 6:**
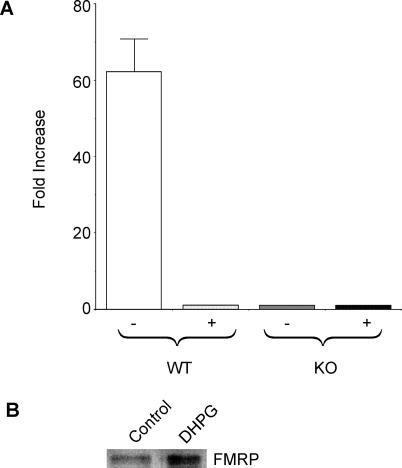
mGluR Activation Dislodges FMRP from APP mRNA (A) APP mRNA was coimmunoprecipitated with FMRP from WT and KO SNs with or without DHPG treatment for 60 min, analyzed by RTqPCR, and plotted as the fold increase in APP mRNA. The data are the average of three experiments. (B) FMRP was immunoprecipitated from WT SNs with or without DHPG for 60 min and analyzed by Western blotting. The data are representative of two experiments.

The mechanism underlying FMRP-mediated translational repression is controversial [36]. Alterations in the association of FMRP with polyribosomes, small nontranslated RNAs, or other proteins have all been proposed [9,12,37,38]. We asked if the APP mRNA/FMRP interaction changed after DHPG. Thus, FMRP was immunoprecipitated from WT SNs (60 min after DHPG), and the pellet was reverse transcribed and analyzed by real-time quantitative PCR (qPCR). APP mRNA was readily detected in anti-FMRP pellets in untreated WT SNs (Figure 6A). However, APP mRNA associated with FMRP could not be detected in DHPG-stimulated WT SN immunopreciptates (IPs) or in the KO with or without DHPG within 40 cycles of real-time PCR. The negative controls for this experiment were duplicate IPs in the absence of 7G1–1 FMRP antibody, which also did not produce any real-time PCR Ct values for APP mRNA within 40 cycles (data not shown). The >60-fold difference in FMRP-associated APP mRNA was highly significant. Evaluation at earlier times revealed that the APP mRNA–FMRP complex was lost within 5 min of DHPG treatment (unpublished data).

Immunoprecipitation of FMRP from WT SNs followed by Western blotting (Figure 6B) or ^35^S-Met incorporation analysis (unpublished data) demonstrated that DHPG treatment does not interfere with the ability of anti–7G1–1 antibody to bind to FMRP. In fact, in both assays there was more FMRP immunoprecipitated from the DHPG-treated WT SNs, which is in agreement with previous reports that DHPG stimulates the dendritic translation of FMRP [22,39]. Our data suggest that physical interactions between FMRP and APP mRNA underlie translational repression, with mGluR activation rapidly moderating these events. Presumably, the loss of FMRP/APP mRNA interaction results in rapid, pulsatile protein expression in dendrites.

### FMRP RNP Binds to G-Rich Sequences

**Figure 7:**
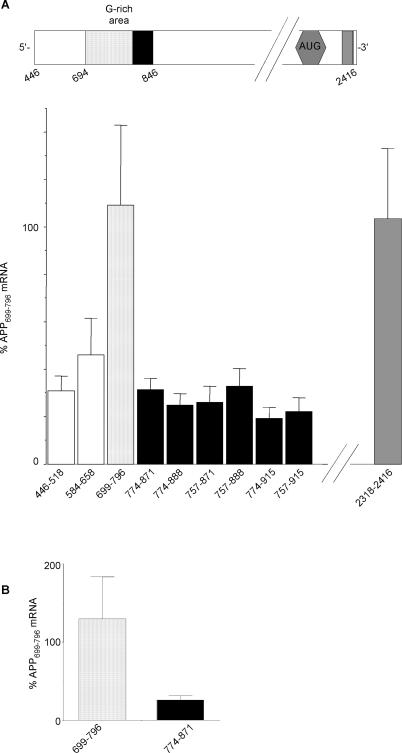
FMRP Binds to a G-Rich Sequence in APP mRNA (A) Relative positions of the G-rich, predicted G-quartet and 29 base elements in nucleotides 446-2500 of APP (top). FMRP IPs digested with ribonuclease T1, analyzed by RTqPCR, and plotted as a percentage of APP mRNA_699–796_ (bottom). (B) FMRP IPs analyzed by the modified CLIP method and plotted as a percentage of APP_699–796_ mRNA.

FMRP is a component of large RNP complexes [38]. The data presented here demonstrate that APP mRNA is also associated with this RNP. To determine the likely interaction site, in vitro RNase protection assays were performed on FMRP IPs from whole-cortex lysates. Residual APP mRNA was mapped by RTqPCR with primers immediately surrounding the predicted G-quartet (Figure 7A). Surprisingly, the G-rich area immediately preceding the G-quartet (nt 699–796) was approximately 4-fold more protected from nuclease digestion than fragments containing the predicted G-quartet (825–846). Although this protected area does not contain a canonical G-quartet motif, the sequence is very G-rich and contains several closely spaced DWGG repeats. The smallest amplicon (nt 774–871) containing the predicted G-quartet motif amplified a 98-base fragment, of which 46 nucleotides were guanines (47% G-rich; Table S1). Although this is the most G-rich amplicon of those tested, and T1 ribonuclease cuts 3′ of single-stranded G-residues, the 98-nt protected fragment (amplicon 699–796) was 40% G-rich, providing nearly equivalent numbers of targets for digestion. Thus, nucleotides 699–796 in the coding region of APP mRNA possess a G-rich sequence that is protected from nuclease digestion by an RNP complex containing FMRP.

The FMRP-containing RNP complex likely protects other *cis*-elements in APP mRNA as well. Our laboratory has defined a 29-base element located 200 nucleotides downstream of the stop codon in APP mRNA that regulates message decay [35]. We have also identified two proteins, nucleolin and hnRNP C, that bind to this 29-base element [40]. Since nucleolin interacts with FMRP to form multiprotein complexes [38], we would expect the 29-base element to be protected from T1 ribonuclease digestion of anti-FMRP IPs, as shown in Figure 7A (nt 2318–2416). Our data suggest that multiple *cis*-regulatory elements of APP mRNA interact with the FMRP-containing RNP complex.

### FMRP Monomer Binds to APP mRNA

Despite the presence of an RNase-protected, G-rich sequence, APP mRNA might not associate directly with FMRP. To determine if this was the case and to further characterize the interaction, we performed a modified CLIP assay [41]. SNs were cross-linked with ultraviolet light, immunoprecipitated with anti-FMRP, digested with T1 ribonuclease, and analyzed by SDS-PAGE. FMRP immunoreactive material (80 kDa) was excised and analyzed by RTqPCR. The amplicon encompassing the G-rich sequence (nucleotides 699–796) of APP mRNA gave a positive signal that was approximately 5-fold greater than that of the predicted G-quartet motif–containing sequence (nt 774–871) immediately downstream (Figure 7B). Thus, our data define the G-rich region immediately preceding the predicted G-quartet as the binding site between FMRP and APP mRNA. The loss of FMRP binding at the G-rich region presumably derepresses APP translation, as it was contemporaneous.

### Soluble Aβ_40_ and Aβ_42_ Are Increased in *fmr-1* KO

**Figure 8:**
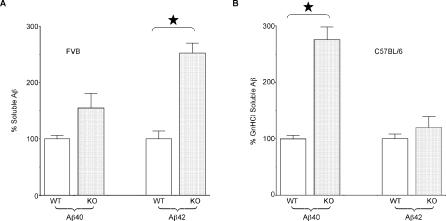
Increased Aβ_40_ and Aβ_42_ Levels in *fmr-1* KO Mice (A) Soluble brain lysates from 1-y-old WT and *fmr-1* KO mice (FVB strain) analyzed by ELISA and plotted as a percentage of soluble Aβ compared to WT controls. Student *t*-tests: *p* = 0.06 (Aβ_40_) and *p* = 0.001 (Aβ_42_). (B) GnHCl-soluble brain lysates from 1-y-old WT and *fmr-1* KO mice (C57BL/6 strain) analyzed by ELISA and plotted as a percentage of GnHCl-soluble Aβ compared to WT controls. Student *t*-tests: *p* < 0.001 (Aβ_40_) and *p* = 0.39 (Aβ_42_).

Increased translation of APP provides more targets for cleavage by β- and γ-secretases. Therefore, we would expect *fmr-1* KO mice to have exacerbated Aβ production with aging. Right-brain hemispheres from middle-aged FVB mice (11–13 mo old) were homogenized in protein extraction buffer containing 1% Triton X-100 and protease inhibitors and the soluble material was analyzed by enzyme-linked immunosorbent assay (ELISA) for Aβ_40_ and Aβ_42_. The *fmr-1* KO mouse brain contained 1.6 times more Aβ_40_ and 2.5 times more Aβ_42_ than WT controls (Figure 8A). We also tested Aβ_40/42_ levels in C57BL/6 mice (12–14 mo old) to ensure that this was not a strain-specific event. We did not observe an increase in soluble Aβ_40_ or Aβ_42_ levels in *fmr-1* KO C57BL/6 brain samples (unpublished data), but guanidine-soluble fractions showed a 2.8-fold increase in Aβ_40_ and a 1.2-fold increase in Aβ_42_ (Figure 8B). Therefore, the brains of two distinct murine strains lacking *fmr-1* both showed increased APP and accumulated pathogenic Aβ species over time.

## Discussion

Synaptic plasticity is required for normal learning and memory and is impaired in FXS. High dendritic spine density is normal for young mice, but synapse pruning during postnatal development is absent in the KO, resulting in increased spine density in adulthood [42]. The molecular basis for defective pruning in *fmr-1* KO mice is unknown, but likely reflects the loss of FMRP-regulated translation of synaptic mRNA. FMRP regulates group 1 mGluR-dependent translation of mRNA targets important in diverse neuronal functions [36]. For example, FMRP normally represses the translation of microtubule-associated protein 1B (MAP1B) mRNA during synaptogenesis. In FXS, MAP1B expression is constitutively elevated, leading to abnormally increased microtubule stability [43]. Therefore, it is of great interest to identify FMRP-dependent synaptic mRNAs that contribute to dendritic structure and function.

Herein, we show that APP mRNA is a previously unappreciated target for FMRP-mediated translational repression at the synapse. The normal physiologic role of APP remains ill defined, but increasing evidence suggests an important role in synapse formation [44,45] and maturation [1]. APP localizes to postsynaptic densities, axons, dendrites, and neuromuscular junctions [1,46]. APP/APP-like protein 2 double-KO mice exhibit defective neuromuscular junctions, excessive nerve terminal sprouting, and defective synaptic transmission [47]. APP is developmentally regulated with maximal expression during synaptogenesis and subsequently declines when mature connections are completed [48,49]. Therefore, synaptic overexpression of APP during early development may contribute to the immature dendritic spines and inadequate synaptic pruning characteristic of FXS.

We have identified a G-rich region located within nucleotides 699–796 in the coding region of APP mRNA as an FMRP-binding site. The G-quartet–like sequence immediately downstream of this G-rich region was not protected from nuclease digestions of FMRP IPs. This result was surprising because the intramolecular G-quartet motif has been identified by in vitro RNA selection assays as the site of interaction with FMRP [17]. As expected from the FMRP interaction site mapping results, alignment of the G-rich region (nt 699–796) and DWGG repeats of mouse APP mRNA is highly conserved with both the human (86%) and rat (93%) sequences. Our data suggest that there may be flexibility in the spacing of the DWGG repeats for G-quartet formation and agrees with previous findings that the presence of a G-quartet does not ensure binding by FMRP [17].

We have determined that FMRP associates with APP mRNA in SN preparations, and that translation of APP mRNA increases in response to DHPG. DHPG-upregulated translation of APP can be blocked by the translational inhibitor anisomycin or the mGluR_5_-specific inhibitor MPEP. mGluR-mediated translation is concurrent with FMRP dissociation from APP mRNA and is independent of mRNA decay. The rapid dissociation of FMRP from APP mRNA, in response to mGluR activation, suggests that post-translational modifications, such as phosphorylation, may regulate FMRP binding activity. Ceman and colleagues have shown that FMRP is phosphorylated N-terminal to the RGG box and that phosphorylation/dephosphorylation status of the protein is correlated with binding to stalled versus active polyribosomes [50]. Our data support a model developing in the literature whereby FMRP acts as an immediate early-response protein that regulates translation at the synapse. When FMRP is bound to APP or other synaptic mRNAs, translation is repressed. Upon mGluR activation, FMRP is released from the nontranslating RNP, resulting in prompt protein synthesis. In FXS, high levels of protein are constitutively produced that are normally translationally repressed by FMRP.

We would predict that constitutively upregulated APP would lead to increased processing to Aβ. Indeed, increased Aβ_40_ and Aβ_42_ are present in two mouse models for FXS. To date, the only abnormal neurpathologic observations in the human FXS brain have involved impaired synaptic pruning and maturation [51]; however, a very limited number of aged FXS brains have been studied [4,29,52], so other neuropathologies, such as increased amyloid burden and synaptic degeneration normally associated with AD, cannot be excluded. It is difficult to measure cognitive decline in mentally retarded individuals; however, in support of our prediction, fragile X–associated tremor/ataxia syndrome in males is associated with dementia [53].

The normal physiologic function(s) of APP are not well understood, albeit the protein is likely important for synapse formation in the developing brain [1]. A recent report demonstrates that children with severe autism and aggression express >2-fold more secreted βAPP (1,200 pg/ml) than children without autism (500 pg/ml) [54]. Many people with FXS (67% of men and 23% of women) are also autistic [55]. Interestingly, the highest levels of secreted βAPP were found in two children with FXS [54]. Thus, overproduction of secreted βAPP may contribute to the developmental disabilities observed in patients with FXS and autism. In addition, FMRP mRNA and protein expression are downregulated as a function of aging in the mouse brain [56], suggesting that repressed transcripts, such as APP, would be upregulated with aging, a well-known phenomenon in animals and humans.

In conclusion, FMRP represses translation of APP mRNA in dendrites, suggesting a link between two neurodevelopmental disorders, FXS and autism, and a neurodegenerative disease, AD.

## Materials and Methods

### Materials.

The anti-FMRP antibody (mAb7G1–1) [10] was obtained from the Developmental Studies Hybridoma Bank, University of Iowa (http://www.uiowa.edu/~dshbwww). The anti-APP polyclonal antibody (catalog number 51–2700) was purchased from Zymed Laboratories (http://www.invitrogen.com), and the anti-mouse β-actin antibody (catalog number A5441), protease inhibitor cocktail (catalog number P2714), ribonuclease T1 (catalog number R1003), and poly(D)-lysine (catalog number P6407) were purchased from Sigma Chemical Company (http://www.sigmaaldrich.com). The anti-rabbit and anti-mouse HRP-conjugated secondary antibodies, percoll, Redivue Pro-Mix-L [^35^S] (catalog number AGQ0080) and enhanced chemiluminescence detection reagents were obtained from Amersham Pharmacia (http://www5.amershambiosciences.com). Anti-22C11 APP antibody (mAB348) was acquired from Chemicon (http://www.chemicon.com). The rabbit polyclonal Aβ_40_ (catalog number 9131), Aβ_42_ (catalog number 9134), and rodent Aβ (catalog number 9154) antibodies were purchased from Signet Laboratories (http://www.signetlabs.com). DHPG (catalog number 0805) was obtained from Tocris Cookson (http://www.tocris.com). Omniscript RT was acquired from Qiagen (http://www.qiagen.com). The MagnaBind Protein A beads, PAGEprep advance kit, and micro BCA protein assay reagent kit were obtained from Pierce Biotechnology (http://www.piercenet.com). DNA oligonucleotides were synthesized by Integrated DNA Technologies (http://www.idtdna.com), and SYBR Green PCR master mix was obtained from Applied Biosystems (http://www.appliedbiosystems.com). NeuroBasal medium, B27 supplement, goat anti-mouse rhodamine-conjugated antibody, and ProLong Gold Antifade with DAPI were from Invitrogen (http://www.invitrogen.com). TRI-Reagent was purchased from Molecular Research Center (http://www.mrcgene.com). MPEP was purchased from Tocris Cookson or synthesized by Technically (http://www.technically.com) and provided by FRAXA Research Foundation (http://www.fraxa.org).

### Mouse husbandry.

The WT and *fmr-1* KO mice in the FVB and C57BL/6 backgrounds were a generous gift from Aaron Grossman and Dr. Bill Greenough (University of Illinois at Urbana-Champaign). The *fmr-1* KO mice were originally developed by Frank Kooy and backcrossed >11 times to FVB mice, albeit these FVB mice have the genes for pigmentation and normal vision [28]. Mice were housed two to four per microisolator cage on a 6am–6pm light cycle with ad libitum food (Purina 5015 mouse diet; http://www.purina.com) and water. The cages contained seeds and a neslet as the only sources of environmental enrichment. All animal husbandry and euthanasia procedures were performed in accordance with the National Institutes of Health and an approved University of Wisconsin–Madison animal care protocol through the Research Animal Resources Center. *fmr-1* genotypes were determined by PCR analysis of DNA extracted from tail biopsies. The FVB strain was used for all experiments described herein except for preparing the cultured neuronal cells (Figure 5) and the Aβ ELISAs (Figure 8B).

### SN preparation and stimulation.

SNs were prepared from WT and *fmr-1* KO mouse cortical tissue [57,58]. Briefly, mouse pups aged 14–17 d were killed by carbon dioxide asphyxiation followed by removal of the brain cortices. The cortices were washed in ice-cold gradient medium (GM buffer: 0.25 M sucrose, 5 mM Tris [pH 7.5], and 0.1 mM EDTA), transferred to a glass dounce homogenizer containing ice-cold GM buffer, and gently homogenized with five strokes of the loose pestle followed by five strokes of the tight pestle. The homogenate was spun at 1000*g* for 10 min at 4 °C in round-bottom tubes to pellet cellular debris and nuclei. The supernatant (2 ml aliquots) was applied to percoll gradients (layers = 2 ml each of 23%, 15%, 10%, and 3% isomotic percoll) and spun at speed (32,500*g*) for 5 min at 4°C. The third band from the top of the gradient (the 23%/15% interface) containing intact SNs was removed and pooled for the experiments. The two higher-molecular-weight bands at the 15%/10% and 10%/3% interfaces contain broken membranes. The salt concentration of the SNs was adjusted by adding one-tenth volume of 10× stimulation buffer (100 mM Tris [pH 7.5], 5 mM Na_2_HPO_4_, 4 mM KH_2_PO_4_, 40 mM NaHCO_3_, 800 mM NaCl). To suppress nonspecific excitation, 1 μM tetrodotoxin was added. The protein concentration of the SNs was determined by Bradford assay and ranged from 200–500 ng/μl.

SNs were equilibrated to room temperature by rotation on a nutator mixer for a minimum of 10 min. DHPG was dissolved in 1× stimulation buffer immediately prior to use and added to the SNs (100 μM final concentration). Samples were mixed at room temperature in 1.5 ml Eppendorf tubes for the indicated times.

### Radiolabeling SNs with ^35^S-Met and immunoprecipitation of APP.

WT and KO SNs (450 μl) were mixed with 25 μl Redivue Pro-Mix-L [^35^S] for 5 min prior to stimulation with 25 μl 2 mM DHPG. Samples were flash frozen at the indicated times. To analyze new protein synthesis, SN lysates were cleared of free isotope, percoll, and sucrose by purification with the PAGEprep Advance kit per the manufacturer's directions. Protein concentrations were determined by the BCA assay, and 15 μg protein was denatured and loaded per lane on 12% SDS gels. The gels were dried and exposed to a phosphorimager screen.

To specifically analyze APP synthesis, WT and KO SN lysates (500 μl) were immunoprecipitated with APP antibody. Briefly, SN lysates were precleared with protein A magnetic beads in 1 ml volumes containing 500 μl SNs, 500 μl 2× IP buffer (20 mM HEPES [pH 7.4], 400 mM NaCl, 60 mM EDTA [pH 8], and 2% Triton X-100), protease inhibitor cocktail, and 100 μl packed fresh protein A magnetic beads. For the immunoprecipitations, 10 μg anti-APP antibody (Zymed catalog number 51–2700) and fresh protein A magnetic beads were added and mixed overnight at 4 °C. The beads were washed three times with IP buffer, and the final, washed pellets were suspended in 40 μl 2× SDS sample buffer and boiled for 5 min; the proteins were then separated on 12% SDS gels. The gels were transferred to nitrocelluose membrane, dried, exposed to a phosphorimager screen, and scanned on a STORM 860 phosphorimager (Molecular Dynamics, http://www6.amershambiosciences.com). The 120-kDa APP bands were quantitated with ImageQuant software (GE Healthcare Life Sciences, http://www4.amershambiosciences.com).

For the inhibitor studies, SNs (425 μl) were preincubated with 25 μl anisomycin (40 μM final concentration) or MPEP (10 μM final concentration) for 10 min prior to the addition of 25 μl ^35^S-Met for 5 min and stimulation with DHPG (100 μM final concentration) for 15 min. Samples were processed as described in the preceding paragraph.

### Western blot analysis.

Aliquots of SNs were collected at 5, 10, and 20 min after DHPG treatment, quenched with an equal volume of 2× SDS sample buffer (8% SDS, 24% glycerol, 100 mM Tris [pH 6.8], 4% β-mercaptoethanol, 0.02% bromophenol blue, 2% Triton X-100, 2% deoxycholate, 2% NP-40 alternative, and 2% sarkosyl) and boiled for 5 min prior to analysis by 12% SDS-PAGE. The separated proteins were transferred to 0.45 μm nitrocellulose membrane in Towbin buffer containing 20% MeOH with a Criterion Blotter (Bio-Rad, http://www.bio-rad.com; 100 V at 4 °C for 75 min). The membranes were blocked in 5% nonfat dry milk and hybridized with anti-rabbit APP antibody (dilution, 1 μg/ml) and anti-mouse β-actin antibody (dilution, 1:20,000) followed by hybridization with anti-rabbit or anti-mouse HRP-conjugated secondary antibodies (dilution, 1:2000). Proteins were visualized by enhanced chemiluminescence on a STORM 860 phosphorimager.

### Neuronal cell culture, confocal microscopy, and image analysis.

Pregnant females (embryonic day 18) were anesthetized with halothane prior to decapitation and transfer of the uterine sac to ice-cold HBSS. Cortices were removed, washed with ice-cold HBSS, lysed with 0.5 mg/ml trypsin for 25 min at 37 °C, washed with HBSS, suspended in NeuroBasal medium (supplemented with 2% B27 supplement, penicillin/streptomycin, and 0.5 mM glutamine), triturated 70× with a 10-ml pipet, and passed through a 70-μm cell strainer. Cells were counted by trypan blue dye exclusion and plated at 1.25 × 10^5^ cells/ml on poly(D)-lysine–coated glass coverslips in 12-well tissue-culture dishes and cultured for 11 d at 37 °C/5% CO_2_. Half of the culture medium was removed and replaced with fresh, warm medium on day 4.

Neuronal cells were treated with 100 μM DHPG, washed with PBS containing 2% FBS, fixed in 4% PHA for 10 min at room temperature, and permeabilized with MeOH (−20 °C) for 15 min. Fixed, permeabilized cells were stained with anti-22C11 against the amino-terminus of APP (Chemicon number mAB348; 1:2000 for 1 h) and visualized with goat anti-mouse rhodamine-conjugated secondary antibody (Invitrogen; 1:500 for 30 min in the dark). All washes and antibody dilutions were in PBS containing 2% FBS. Coverslips were fixed to slides with 12 μl ProLong Gold Antifade with DAPI (Invitrogen) and dried overnight.

Images were acquired with a Nikon C1 laser-scanning confocal microscope with EZ-C1 v2.20 software (Nikon, http://www.nikon.com) at 60× magnification. APP levels in the puncta of four to seven dendrites per sample were quantitated with IMAGE J software using the Analyze Particles function (minimum of 205 puncta analyzed per treatment) (Rasband, W.S., Image J, U.S. National Institutes of Health, http://rsb.info.nih.gov/ij; 1997–2006). Figures were assembled with Adobe Photoshop 8.0 (Adobe Systems, http://www.adobe.com). All DHPG-treated and *fmr-1* KO samples were highly statistically different from untreated WT samples by *t*-test analyses (*p* <0.001) and expressed as SEM.

### APP mRNA measurements.

Aliquots of SNs were collected at the indicated timepoints and flash frozen at −80 °C. The samples were thawed and vortexed to prepare SN lysates. To directly reverse-transcribe RNA from SN lysates without an RNA purification step, a modified method for the detection of mRNA in single neurons was utilized [59]. Briefly, 2 μl SN lysate was added per standard RT reaction containing RNase-free DNase I and random nonamer primer. The reactions were incubated at 37 °C for 15 min to destroy any contaminating genomic DNA, 65 °C for 5 min to inactivate the DNase I, and 20 °C for 10 min to anneal the random primer. Omniscript RT was added and reverse transcription proceeded at 37 °C for 60 min before inactivation at 93 °C for 5 min. The RT reactions were diluted 5-fold with DEPC water prior to real-time PCR analysis. For the statistical analysis, APP mRNA levels from triplicate experiments were determined, normalized to 18S rRNA, and plotted as a percentage of total APP mRNA. Error bars depict SEM.

### Real-time PCR controls, standard curves, and analyses.

The PCR primers were designed with Primer Express software from Applied Biosystems, and BLAST homology searches of the amplicons revealed that the primers were gene specific. PCR reactions were optimized for primer and template concentrations and contained 500 nM APP primers (forward: 1701-ccgtggcacccttttgg-1717; and reverse: 1774-gggcgggcgtcaaca-1760) or 300 nM 18S primers (forward: 98-cattaaatcagttatggttcctttgg-123; and reverse: 181-tcggcatgtattagctctagaattacc-155), 10.5 μl 1:5 diluted RT reaction and 12.5 μl SYBR green PCR mix in a 25 μl reaction volume. The cycle conditions were as follows: 2 min at 50 °C and 10 min at 95 °C (40 cycles: 15 s at 95 °C, 1 min at 60 °C), followed by a dissociation stage for 15 s at 95 °C, 1 min at 60 °C, and 15 s at 95 °C. The average PCR efficiencies for the APP and 18S primers over a 200-fold concentration range were 100% (APP) and 101% (18S) (*n =* 9 experiments each), with a delta slope of 0.079. As the difference in slopes between the sample PCR (APP) and the normalization control (18S) was less than 0.1, the comparative C_T_ method was utilized to calculate the relative concentration of APP mRNA normalized to 18S rRNA. SNs are void of nuclei; however to ensure there was no genomic DNA contamination, control RT reactions on SN templates in the absence of reverse transcriptase were analyzed by real-time PCR and found void of APP PCR product. The final APP and 18S PCR products were analyzed by agarose gel electrophoresis and were single bands of the correct molecular weight by EtBr staining (74 bp for APP; 84 bp for 18S).

### FMRP IPs and real-time PCR analysis.

SN lysates were precleared with protein A magnetic beads and immunoprecipitated with 10 μl RNasin, 10 μg 7G1–1 FMRP antibody (or no antibody controls), and 100 μl packed fresh protein A magnetic beads for 3 h at 4 °C. The IPs were washed with IP buffer (10 mM HEPES [pH 7.4], 200 mM NaCl, 30 mM EDTA [pH 8], and 0.5% Triton X-100) and suspended in 1 ml TRI-Reagent. Total RNA was isolated and precipitated in the presence of 2 μg tRNA. The final pellet was suspended in DEPC water, solubilized 10 min at 60 °C, and reverse transcribed with Qiagen Omniscript and random nonamer primer (60 min at 37 °C, 5 min at 93 °C). The cDNA was diluted 5-fold and analyzed for APP by qPCR as described immediately above.

### Preparation of whole-cortex lysate.

The cortices from six WT mice (13 d old) were torn into pieces and homogenized in cold immunoprecipitation buffer (10 mM HEPES [pH 7.4], 200 mM NaCl, 30 mM EDTA [pH 8], and 0.5% Triton X-100) containing 2× protease inhibitor cocktail and 0.4 U/μl RNasin. The homogenate was spun at 1,000*g* for 10 min at 4 °C to remove nuclei and unlysed cells, and the pellet was discarded. The cleared lysate was flash frozen in aliquots at −80 °C.

### Ribonuclease TI digestions and modified CLIP assay.

Pellets from anti-FMRP immunoprecipitations of whole-cortex lysate were washed once with immunoprecipitation buffer and once with DPBS before digestion with ribonuclease TI (0.8–4.0 U) in a 100-μl reaction volume for 30 min at 37 °C with occasional mixing to disperse the magnetic protein A beads. The digested samples were washed twice with DPBS to remove RNA fragments. Protected RNA was isolated with TRI-Reagent and analyzed by RTqPCR. The primer sequences for the real time PCR are listed in Table S2. The delta C_t_ between undigested and T1-digested samples was calculated and plotted as a percentage of APP_699–796_ mRNA.

For the modified CLIP assay [41], cleared cortical lysate was cross-linked with 400 mJ/cm^2^ ultraviolet light in an UV Stratalinker 2400 (Stratagene, http://www.stratagene.com), immunoprecipitated with anti-FMRP, and digested with ribonuclease TI. The washed pellets were suspended in 40 μl SDS loading buffer containing no reducing agents, heated for 10 min at 70 °C, applied to 12% SDS/PA gels, and transferred to 0.45 μm nitrocellulose membrane in Towbin buffer. Western blotting of a duplicate membrane indicated that FMRP migrates at 80 kDa. A band encompassing approximately the 75–85 kDa molecular weight range was excised, transferred to TRI-Reagent, and vortexed vigorously for 15 min at 37 °C. RNA was isolated and analyzed by RTqPCR.

### Aβ_40_ and Aβ_42_ ELISAs.

For soluble brain lysates, right hemispheres from four WT (aged 11, 13, 13, and 13 mo) and three KO mice (aged 11, 12, and 12 mo; FVB strain) and four WT (aged 13.5, 13.5, 12, and 12 mo) and four KO mice (aged 14, 14, 12, and 12 mo; C57BL/6 strain) were homogenized in 500 μl protein extraction buffer (10 mM Tris [pH 7.6], 2 mM EDTA, 150 mM NaCl, 1% Triton X-100, 0.25% NP-40, and 1× protease inhibitor cocktail). Insoluble material was removed by centrifugation at 12,000 rpm for 10 min, and aliquots of the soluble fraction were flash frozen. For total brain lysates, left hemispheres were homogenized in cold, 5 M GnHCl, mixed for 3–4 h at room temperature, and frozen at −80 °C. Sandwich ELISAs with the Signet Aβ_40_/9131 and Aβ_42_/9134 capture antibodies and the rodent Aβ/9154 reporter antibody were performed as previously described [60]. Aβ levels were quantified based upon standard curves run on the same ELISA plate and then expressed as a percentage of Aβ compared to WT controls.

## Supporting Information

Figure S1 G-Quartet Model A model for the putative G-quartet sequence located in the coding region of APP mRNA. Canonical G-quartets form two guanine planes, while the putative APP G-quartet has the potential to form three guanine planes.
(46 KB TIF)

Figure S2 mGluR-Dependent APP Translation Is Independent of mRNA Decay WT and KO SNs with or without DHPG (5, 10, 15, and 30 min) were analyzed by RTqPCR. APP mRNA levels were normalized to 18S rRNA and plotted as a percentage of total APP mRNA.
(166 KB TIF)

Table S1 G-Richness of APP Amplicons (15 KB XLS)

Table S2 Real-Time PCR Sequencing Primers (16 KB XLS)

### Accession Numbers

The GenBank (http://www.ncbi.nlm.nih.gov/Genbank) accession numbers for the gene products mentioned in this paper are human APP mRNA (NM_000484), mouse APP mRNA (X59379), rat APP mRNA (X07648), and 18S mRNA (M27358).

## Abbreviations

AD: Alzheimer disease
APP: amyloid precursor protein
Aβ: beta-amyloid
ELISA: enzyme-linked immunosorbent assay
FMRP: fragile X mental retardation protein
FXS: fragile X syndrome
IP: immunoprecipitate
KO: knockout
mGluR: metabotropic glutamate receptor
PSD-95: postsynaptic density 95 protein
RNP: ribonucleoprotein
RTqPCR: real-time quantitative PCR
SN: synpatoneurosome
